# Influence of Physical Fitness and Attention Level on Academic Achievements of Female and Male Military Academy Cadets in Poland

**DOI:** 10.3390/healthcare9101261

**Published:** 2021-09-24

**Authors:** Dariusz Jamro, Grzegorz Zurek, Maciej Lachowicz, Dariusz Lenart

**Affiliations:** 1 Department of Physical Education and Sport, General Tadeusz Kosciuszko Military University of Land Forces, 51-147 Wroclaw, Poland; dariusz.jamro@awl.edu.pl (D.J.); dariusz.lenart@awl.edu.pl (D.L.); 2 Department of Biostructure, Wroclaw University of Health and Sport Sciences, 51-612 Wroclaw, Poland; maciej.lachowicz@ideis.pl

**Keywords:** physical fitness, academic achievements, attention, army cadets, women

## Abstract

A professional soldier should be characterized not only by high physical fitness but also by high intellectual and cognitive skills. Therefore, it is important to focus on the future commanding cadre-cadets that are educated in military academies. The aim of the study was to look for correlations between the level of physical fitness and attention and academic achievements (AA) in different subjects among cadets studying at military academies. The research group consisted of students of a military academy in Poland, i.e., 228 cadets, including 31 women and 197 men. Correlations between explained and explanatory variables were assessed using Pearson’s correlation. Correlations between AA and somatic parameters and motor components were analysed using optimal regression, using the best subset method. A statistical difference was found between men and women in terms of the level of somatic and motor development; men also performed significantly better in practical military subjects. In the female group, dominant hand strength is a significant determinant of AA in civilian and theoretical military subjects. The findings suggest the need for specialized training aimed at bridging the major differences in physical fitness between men and women by placing greater emphasis on muscle strength development.

## 1. Introduction

High physical fitness has always been perceived as an indispensable attribute of a soldier. It is still the basis for assessing the combat value of the modern army. Despite the progressive civilizational development, digitalization and robotization, military service will always be associated with high physical fitness.

The most important goal of physical fitness is positive physical health, which determines the low risk of health problems and also the development of the ability to engage in daily tasks with adequate energy [1].

With the current knowledge, there is no doubt that physical activity has many health benefits. It is an important protective factor against many health disorders and chronic non-infectious diseases such as obesity, type 2 diabetes, cardiovascular diseases (hypertension, cardiac arrest) and age-related diseases such as dementia and Alzheimer’s disease [2]. The risk of diseases such as osteoporosis and ischemic heart disease in adulthood can be reduced by regular physical activity from an early age [3,4,5].

In addition, researchers have confirmed the positive effects of exercise on cognitive function due to increased blood flow to the brain; more neurotransmitters; improved plasticity, focus and attention; and more efficient information processing [6,7,8,9]. More frequent physical activity is associated with higher levels of cognitive processing by increasing control over these processes [10], and early intervention to support the uptake of physical activity may be important for maintaining cognitive health, particularly in adulthood and old age [7].

The literature on the links between physical fitness and learning performance is quite extensive. Studies support a positive correlation between physical fitness and academic achievement and its impact on some cognitive functions [11,12,13,14,15]. Examination scores and grades during learning, directly representing the level of cognitive functions, significantly correlate with physical fitness [16,17,18]. Physical fitness is positively correlated with academic achievement and also negatively correlated with BMI [19]. Physical activity is positively related with cognitive function [20,21,22,23].

In a study by Bezold et al. [24], an increase in physical fitness level over the course of a year of schooling led to higher academic achievement achieved by students compared to other subjects whose fitness level did not change. At the same time, a decrease in physical fitness level was associated with lower academic performance.

Implementing an adequate amount of physical exercise during childhood may ensure better cognitive fitness that translates into, among other things, better academic performance, especially in a risk group such as those who are prematurely born [25].

A low level of cardiorespiratory fitness combined with low cognitive fitness in early adulthood results in a higher risk of dementia and mild cognitive impairment in later life [26]. Attention to cardiorespiratory fitness may help to reduce the deleterious effects of aging on brain structures [27].

Physical exercise can be an important instrument in public health efforts, as well as in projects that optimize educational achievement, cognitive abilities, and prevention efforts against civilization diseases at the community level [28].

With the current state of knowledge, it is obvious that processes related to attention, concentration and other executive functions are linked to learning performance reflected in academic performance. Recent studies support these correlations [29,30,31,32]. Most studies have been conducted in clinical settings. However, only a few publications have addressed attention and executive functions among academic adolescents without developmental problems. There is also a lack of such studies among cadets. Therefore, our own research was enriched by conducting the adult version of the Color Trail Test (CTT).

The CTT is a cross-cultural version of the neuropsychological Trail Making Test (TMT) that minimizes the impact of language differences. It examines the efficiency of processes related to attention and executive functions (sequencing, mental flexibility, visual search as well as motor skills). For our study, we used the first and second part of the Color Trail Test, which are equivalents of the TMT Part A and Part B [33].

The dynamics of changing conditions on the battlefield, the constant flow of information and the need to make the right decision, often under a time regime, require from a soldier (and especially from a commander-leader) extremely highly developed cognitive functions. The specific nature of military service places before soldiers the expectations of rapid learning, efficient assimilation of specialized knowledge and also operating in practice of specific psychomotor skills.

The research also focused on the military service of women. Relatively recently, they have been permitted to study in military academies. There are currently 917 women studying in Poland. Normative documents give female and male soldiers the same rights and impose the same obligations [34].

Researchers addressing the issue of women’s physical fitness in the military have demonstrated, among other things, that female soldiers experience a greater physiological load when performing the same operational (physical) tasks as men. During military exercises, women are at greater risk for musculoskeletal injuries. This may be due to the need to exert relatively more effort to perform psychomotor tasks at a similar level as men [35,36].

There is a lack of research in the military academies addressing the links between physical fitness and attention with intellectual fitness as manifested in academic achievement, among other things. This problem seems to be particularly relevant at the stage of training future commanders.

The aim of this study is to search for the correlation between the level of physical fitness and attention and learning performance in various subjects of female and male cadets studying at the military academy.

## 2. Materials & Methods

In order to find relationships between physical fitness and attention levels and AA in different subjects among female and male cadet groups, we designed a cross-sectional study. This allowed us to examine the hypothesized relationships between the study variables after the first semester of studies.

The study was based on the consent of the Rector-Commandant of the Military University of Land Forces (no. 271 dated 18 January 2021) and the approval of the Research Ethics Committee of the Wroclaw University of Health and Sport Sciences (no. 2/2021 dated 12 February 2021). Recruitment for the study took place on 22 February 2021, which included an organizational meeting, presentation of the study design and submission of conscious consent to participate in the study. All data were collected in February 2021 at the Military University of Land Forces in Wroclaw.

The study group consisted of students from the Military University of Land Forces in Wroclaw who were admitted to the first year of studies in the field of Commanding in the academic year 2020/2021—that is, 228 cadets, including 31 women (age = 20.91, SD = 1.19) and 197 men (age = 20.66, SD = 1.30).

To determine the level of physical fitness, measurements and tests of individual fitness components were conducted according to the concept of health-related fitness (H-RF):

1. Anthropometric measurements

Anthropometric measurements (body height and mass) were performed in sportswear in the sports hall. Height (accuracy of measurement 1 mm) and body mass (accuracy of measurement 0.1 kg) were measured using the Martin technique with a medical anthropometer SECA. Based on the measured somatic parameters, body mass index (BMI) was calculated. Basic data on age and sex of the subjects were obtained from a questionnaire.

2. Motor components

Motor components were assessed by measuring static hand strength and speed (10 × 5 m shuttle run). The examination was preceded by a warm-up, demonstration and verbal commentary, according to the guidelines of “Eurofit for adults” [37].

a. Dominant hand strength

Hand strength was measured using a Stanley hydraulic hand dynamometer with a measurement accuracy of 1 kg. The subject, holding the dynamometer in the fitter hand away from the torso, clenched the hand as hard as they could. The pointer on the dynamometer stops at the highest reading until it is zeroed. The test was performed twice, with the better result used for analysis.

b. Running speed

The subject stood in a starting position with both of their feet in front of the line. After the command “START”, they ran as fast as they could to the second line 5 m away, crossed it with both feet, and came back. During the run, and especially during the turns, the subjects were not allowed to support themselves with hands on the floor. The test was performed once.

3. Attention

Processes related to attention and some executive functions were examined using the Color Trails Test (CTT) in CTT-1 and CTT-2 versions.

The test is performed on an A4 sheet on which there are pink and yellow numbered circles. For CTT-1, the respondent uses a pencil to connect consecutively numbered circles from 1 to 25 as quickly as possible, while for the second part (CTT-2), the respondent connects consecutively numbered circles alternating between pink and yellow as quickly as possible. The time of completion of each trial and qualitative performance characteristics are recorded.

The tests were conducted and checked by Dariusz Jamro, M.Sc., an academic lecturer at the Military University of Land Forces who has the necessary skills in the research tool used. Measurements were made in accordance with all the requirements [33]. Measurement errors may have occurred during the validation of the test. No errors are noted in the application of the tool itself in a population without cognitive health problems.

4. Academic Achievements
  AAs were determined by the arithmetic mean of grades in the subjects:
  c. Civil subjects: humanities and social subjects (Polish history, English language, basics of management) and science and natural sciences (computer science in command, mathematics);
  d. Theoretical military subjects (shaping patriotic attitudes, security theory and classification of threats);
  e. Practical military subjects (physical education, shooting training, military topography).

Physical fitness tests and anthropometric measurements were always performed at the same time of day and in the same place. The warm-up always had the same form. The CTT tests were performed on a single day in a lecture hall with a thorough explanation of how to perform the test. The correctness and evaluation of CTT performance was checked twice. Data were collected by the physical education staff of the Department of Physical Education and Sports at the Military University of Land Forces specialized in scientific research.

Quantitative variables in the form of physical fitness components (age, weight, height, BMI, handgrip strength, running speed and agility) and attention-related variables (CTT-1 and CTT-2) were treated as explanatory variables in this study. Quantitative variables in the form of academic achievement (mean grades in each group of subjects) were the explained variables.

## 3. Statistical Analysis

The collected results were subjected to detailed statistical analysis by calculating for all variables: normality of the distribution of individual variables was assessed using the Shapiro–Wilk test (for the group of women, N = 31) and the Kolmogorov–Smirnov test (for the group of men, N = 196), relationships between explained and explanatory variables were assessed using Pearson’s correlation and relationships between AA and somatic parameters and motor components were analysed using optimal regression, using the best subset method. The condition of normal distribution was met in order to apply the method of optimal regression. This made it possible to determine the optimal set of explanatory variables (taking into account the interdependencies between them) that could best predict the performance of the explained variable. We decided to include four variables in each model because of the different types of variables analysed in the article—(1) anthropometric variables: age, body height, body mass; (2) strength as a component highly desirable for military service; (3) speed as a component related to cognitive speed; and (4) variables related to attention level: CTT-1 and CTT-2. The authors subjectively expected that each type of variable could enter the model. Statistical significance in the study, for all tests used, was taken at *p* < 0.05.

All calculations were performed in a professional program Statistica v. 13 from StatSoft Poland in the Department of Biostructure of the Wroclaw University of Health and Sport Sciences, certified by ISO 9001.

## 4. Results

Among the somatic traits and motor components, significantly higher scores were registered in males (Table 1). In contrast, females obtained higher results in a test checking the level of attention and some executive functions, with statistically significantly better results in CTT-2 (61.97 ± 11.94, *p* = 0.0230). Differences in CTT-1, although noticeable, also turned out not to be statistically significant in favour of females (30.87 ± 9.44, *p* = 0.0748) (Table 1).

Among the analysed groups of subjects, significant differences occurred in the results of civilian (science and natural sciences) and military (practical) subjects. Women performed better in civilian subjects (science and natural sciences) (4.02 ± 0.21, *p* = 0.0155), while men showed better results in military (practical) subjects (4.20 ± 0.30, *p* = 0.0003). The differences in AA in the other groups of subjects, i.e., civil subjects (humanities and social sciences) and military subjects (theoretical), were not statistically significant.

The analysis of simple correlations between the analysed somatic and motor variables, level of attention, and AA allow us to state that in the female group, the correlations are partially statistically significant (Table 2). Achievement in civil subjects (humanities and social sciences) and CTT-1 test scores appeared to be positively correlated with dominant hand strength.

The highest correlations were found in the results obtained in civil subjects (sciences and natural sciences). They correlated positively with body weight, body mass index BMI and right-hand grip strength.

In the analysis of the correlations between components of physical fitness, attention level and AA in the male group, there are essentially no correlations (Table 3). Dominant hand strength was positively associated with performance in civilian subjects (science and natural sciences). In contrast, higher scores in military (theoretical) subjects were favoured by lower hand strength. Men with greater body height achieved better results in military (practical) subjects; however, the strength of this correlation was very weak.

The results of the regression analysis show that the strength of the dominant hand turned out to have the greatest influence on AA in civilian and military (theoretical) subjects in the female group; these relationships were revealed in the analysis of simple correlations as well as in the regression analysis (Table 2 and Table 4). In this case, it is a strong determinant of AA in civilian and military (theoretical) subjects, which can explain, together with the CTT-1 attention test, 30.7% of the variation in civilian (humanities and social sciences) results; together with body mass and height, 43.2% of the variation in civilian (science and natural sciences) results; and together with BMI, body mass and height, 35.5% of the variation in military (theoretical) results. The appearance of body mass in the regression models, on the other hand, seems simple to explain, as many studies show a relationship between static strength and body mass, which is, after all, the biological basis of this ability.

In the male group, the result of regression analysis showed only one statistically significant model. Academic achievement in military theoretical subjects was favored by lower hand grip strength, higher body mass index, lower body weight, and higher body height. In the model, these variables collectively explained 6.5% of the variability in performance in military theoretical subjects.

## 5. Discussion

A future professional soldier should be characterized not only by high physical fitness but also by high intellectual and cognitive skills. The aim of the training of cadets at the Military University of Land Forces is for graduates to have the ability to recognize, diagnose and solve problems while commanding a subunit, including leadership, education, training, methodology, managing human resources, effective communication, as well as negotiation and responsibility for commanding and training subordinates.

The main results of our study are the results of correlation and regression analysis in the female group, indicating that the level of physical fitness (mainly the strength component) but also partially the level of attention, body mass and BMI influenced the academic achievements of female cadets. Strength level significantly positively correlated with performance in three of the four subject groups analysed. Additionally, hand muscle strength entered three significant models with a high adjusted R^2^ fit coefficient, explaining the variability in academic achievement. It should also be noted that in the male group, the result of regression analysis showed only one statistically significant model. Academic achievement in military theoretical subjects was favored by lower hand tightness, higher body mass index, lower body weight and higher body height. Of course, this was not the expected result; however, the coefficient of determination of this model was only 6.5%, which may indicate randomness, and it is difficult to predict academic achievement from such a result.

With reference to attention, variables measured by the CTT-1 and CTT-2 instruments did not influence the theoretical and practical military competencies. The study was conducted among a heavily selected group of individuals in terms of school performance. Probably because of this, the desired results were not obtained in this area of research.

Additionally, in our research, the results of motor tests indicate a significant advantage of men over women in the level of strength. This is of great practical significance, as in specific military tasks (marching with loads, lifting and transporting heavy military equipment including weapons, ammunition, mines, machines, etc.), strength has a direct impact on the quality of performed tasks. Women are much less able to perform tasks involving the use of force, especially when it is necessary to use force for a longer period. Moreover, as a result of strength-intensive activities, women suffer numerous injuries, muscular pains, and overloading of, e.g., the knee joints and the lumbar spine [35,36,38,39,40].

With reference to the above results, women performed significantly better in civilian subjects (sciences and natural sciences) (4.02 ± 0.21, *p* = 0.0155), while men achieved significantly higher results in military (practical) subjects (4.20 ± 0.30, *p* = 0.0003). It is not surprising that men outperformed women in the group of military (practical) subjects, the results of which were directly related to subjects (physical education, shooting training, military topography) requiring physical activity and therefore are directly favored by higher physical fitness.

With the current state of knowledge, the advantage of men over women in muscle strength is well known and results, among others, from a higher accumulation of testosterone, causing the growth and development of muscle mass during sexual maturation in men. Additionally, in muscle building, men have a predominance of type II fast-twitch muscle fibres, as well as a greater ratio of type II muscle fibres to type I. Muscle strength is also associated with somatic development—greater body height and muscle mass, which were characteristic of the men studied [36,40].

Arm strength, which is a measure of the strength of the examined men, is particularly important because its high level is extremely desirable in candidates for professional soldiers. Already at the recruitment stage to military academies in Poland, strength tests are an inseparable element of physical fitness tests for both men and women. Then, in the course of military studies, semester credits include strength tests, starting with hanging on a bar with arms bent, through marching and running with loads and ending with pull-ups on a bar and instrumental gymnastics. An adequate level of strength is a desirable motor ability that has been the subject of much research at other military academies and cadet training centres around the world [41,42,43,44,45]. The presence of strength in the regression analysis can be explained by the need for women to undertake strength training, as they are forced to put the most work into shaping their strength to meet the demands of daily military service. Equally important is one of the didactic rigours required to pass after each semester of military studies, i.e., pull-ups on a bar.

It is interesting to note that the group of subjects whose achievements were affected by strength included only subjects of a theoretical nature, i.e., subjects that are relatively easy to learn compared to practical military subjects. This can be explained by the initial stage of the subjects’ training, where practical military skills are only just being taught and require distinctly different competencies than theoretical military or civilian subjects. Therefore, it can be assumed that the impact of physical fitness on practical military subjects may become apparent in the following years of schooling. A breakthrough in this respect may turn out to be the second year of education when a specialist module begins containing a significant number of military specialist subjects, mainly practical ones.

Academic achievements analysed in the study directly represent the level of cognitive functions of cadets, which significantly correlated with physical fitness [16,17,18]. In our study, the level of attention examined by the CTT-1 test significantly correlated with academic achievements in military (theoretical) subjects in a group of women. The results of studies available in the literature on the relationship between physical fitness and cognitive functions, cited in this paper, concerned mainly soldiers conscripted or in the private corps [25,26,28]. There is a lack of this type of research in the military higher education environment.

Despite the wealth of information on physical fitness and the key role it plays in military life, there is a lack of work in the professional soldier community and particularly in military academies on the relationship between the level of motor skills, somatic development, intellectual fitness and academic and military achievements.

Researchers in military academic centres continuously focus their attention mainly on motor skills, sometimes neglecting the cognitive sphere. This problem should be looked at in the context of specific tasks and competencies required for a given service position in the army. While for a private soldier, the basis is physical fitness, which is needed to perform the basic combat tasks involving mainly highly developed motor components, an officer or commander, in addition to equally high physical fitness, requires above-average cognitive fitness—creativity, focus, multi-tasking, quick thinking, learning, etc. This fitness can be manifested in a variety of ways, such as: the ability to perform a specific task, the ability to think and learn quickly, the ability to use a variety of methods, and the ability to use a variety of tools. This fitness may be manifested in cognitive activities in which the quality of a decision concerning the execution of a task is important; an example may be a decision when choosing a way to attack an enemy object on the battlefield.

There are some limitations that should be considered when interpreting the results of this study. First, the study was conducted among a heavily selected group of individuals in terms of physical fitness and school performance. In order to be admitted to a military academy, cadets had to have high scores on their high school final exams and go through a demanding admissions process, including physical fitness exams and an interview. Therefore, the results of the study should not be generalized to all students. Moreover, the results of such a selected group of individuals may often not provide the expected results.

Another limitation is that the study was conducted in the early stages of training, at the end of the first semester of study, where differences in physical fitness levels in the context of academic achievement may not yet become apparent. Additionally, as a cross-sectional study, they cannot describe or explain the cause–effect correlation between physical fitness level and attention and cadets’ academic achievement. Therefore, the above study will be continued on the same group of subjects in further years of education by the authors of this paper.

In addition, important individual-level factors that may be responsible for part of the correlations between physical fitness and academic achievement, such as cadets’ motivation, self-control, individual aptitudes and interests, intelligence level or finally social factors, were not measured.

The small number of women (N = 31) compared to the men participants (N = 197) should also be noted; however, the study group consisted of all cadets enrolled in the first year of study.

## 6. Conclusions

The results of the research will be used to introduce specialized training aimed at eliminating the biggest differences in physical fitness between men and women by putting more emphasis on the development of strength. Examples include additional physical education classes and training for women only, such as circuit training in the gym with their own body weight, marching with weights, overcoming obstacle courses, and CrossFit. Organized exercises to prevent injuries and trauma-stretching, flexibility and stabilization exercises may also be beneficial. It should be expected that it will result in optimal performance of tasks by both genders during joint tactical exercises in military life [35,36].

Conducting further research among female and male cadets may translate into the optimization of the training process in military academies, which play a significant role in national defence. Moreover, the results of the study will certainly provide further valuable data for researchers dealing with broadly defined physical and mental health.

## Figures and Tables

**Table 1:** Variation in mean values of morphological and motor characteristics as well as AA and attention level by Student’s *t*-test for independent samples between men and women.

Variable	Men (N = 197)			Women (N = 31)			*T*-test		g
	$\bar{x}$	sd	v	$\bar{x}$	sd	v	*t*	*p*	
Age [years]	20.66	1.30	6.27	20.91	1.19	5.70	−1.02	0.3076	0.19
Body height [cm]	179.38	5.66	3.15	167.67	4.04	2.41	11.07	**0.0000**	2.14
Body mass [kg]	77.69	7.86	10.12	63.37	4.49	7.09	9.88	**0.0000**	1.90
BMI [kg/m^2^]	24.13	2.06	8.54	22.50	1.39	6.19	4.23	**0.0000**	0.82
Strength of the dominant hand [kg]	124.62	18.73	15.03	88.87	13.77	15.49	10.20	**0.0000**	1.97
Shuttle run 10 × 5 m [s]	18.45	1.02	5.53	19.63	0.83	4.22	−6.12	**0.0000**	1.18
CTT-1 [s]	33.96	8.87	26.11	30.87	9.44	30.58	1.79	0.0748	0.34
CTT-2 [s]	68.42	14.96	21.86	61.97	11.94	19.27	2.29	**0.0230**	0.31
Civil subjects (humanities and social sciences) [grade]	4.40	0.29	6.63	4.46	0.33	7.35	−0.93	0.3529	0.20
Civil subjects (science and natural sciences) [grade]	3.90	0.27	6.96	4.02	0.21	5.16	−2.44	**0.0155**	0.46
Military subjects (theoretical) [grade]	4.70	0.26	5.61	4.73	0.25	5.35	−0.52	0.6005	0.12
Military subjects (practical) [grade]	4.20	0.30	7.13	3.98	0.36	9.06	3.65	**0.0003**	0.80

$\bar{x}$ —mean, sd—standard deviation, v—coefficient of variation, g—Hedges’ g effect size. Bold indicates *p* < 0.05.

**Table 2:** Correlations between the studied characteristics in the female group. Bold, highlighted correlation coefficients are significant with *p* < 0.05.

Variable	95% CI	Age	Body Height	Body Mass	BMI	CTT-1	CTT-2	Hand Grip	Shuttle Run 10 × 5 m
Age	20.47–21.35	-	0.10	0.13	0.05	−0.08	0.03	0.21	−0.15
Body height	166.19–169.16	0.10	-	**0.57**	−0.25	−0.05	0.05	0.23	−0.05
Body mass	61.72–65.02	0.13	**0.57**	-	**0.64**	0.22	0.28	**0.52**	−0.19
BMI	21.99–23.01	0.05	−0.25	**0.64**	-	0.34	0.32	**0.38**	−0.20
CTT-1	27.41–34.33	−0.08	−0.05	0.22	0.34	-	**0.63**	−0.15	**0.41**
CTT-2	57.59–66.35	0.03	0.05	0.28	0.32	**0.63**	-	−0.26	0.26
Hand grip	83.82–93.92	0.21	0.23	**0.52**	**0.38**	−0.15	−0.26	-	−0.14
Shuttle run 10 × 5 m	19.33–19.93	−0.15	−0.05	−0.19	−0.20	**0.41**	0.26	−0.14	-
Civil subjects (humanities and social sciences)	4.34–4.58	0.01	0.20	0.31	0.19	0.11	−0.22	**0.52**	−0.11
Civil subjects (science and natural sciences)	3.95–4.10	0.29	−0.08	**0.43**	**0.57**	0.04	−0.02	**0.55**	−0.26
Military subjects (theoretical)	4.63–4.82	0.18	0.16	0.14	0.07	−0.30	−0.29	**0.53**	−0.21
Military subjects (practical)	3.85–4.12	−0.08	−0.05	−0.27	−0.25	−0.18	−0.27	0.02	−0.10

CI—confidence interval, bold indicates statistical significance (*p* < 0.05).

**Table 3:** Associations between the studied characteristics in the male group. Correlation coefficients highlighted in bold are significant with *p* < 0.05.

Variable	95% CI	Age	Body Height	Body Mass	BMI	CTT-1	CTT-2	Hand Grip	Shuttle Run 10 × 5 m
Age	20.48–20.84	-	0.02	0.10	0.08	−0.01	0.07	0.00	0.11
Body height	178.59–180.18	0.02	-	**0.54**	−0.12	0.07	−0.09	0.10	−0.01
Body mass	76.59–78.80	0.10	**0.54**	-	**0.76**	0.10	−0.07	0.13	0.03
BMI	23.84–24.41	0.08	−0.12	**0.76**	-	0.06	−0.03	0.08	0.05
CTT-1	32.72–35.21	−0.01	0.07	0.10	0.06	-	**0.42**	0.02	−0.01
CTT-2	66.32–70.52	0.07	−0.09	−0.07	−0.03	**0.42**	-	0.10	−0.01
Hand grip	121.99–127.26	0.00	0.10	0.13	0.08	0.02	0.10	-	**−0.19**
Shuttle run 10 × 5 m	18.31–18.59	0.11	−0.01	0.03	0.05	−0.01	−0.01	**−0.19**	-
Civil subjects (humanities and social sciences)	4.36–4.44	−0.13	0.10	0.06	0.00	−0.06	−0.05	−0.06	0.04
Civil subjects (science and natural sciences)	3.86–3.94	0.03	−0.07	−0.01	0.04	−0.05	0.06	0.12	−0.07
Military subjects (theoretical)	4.66–4.74	0.03	0.10	0.06	0.01	0.04	0.06	**−0.19**	0.04
Military subjects (practical)	4.16–4.24	0.06	**0.17**	0.14	0.04	0.03	−0.06	0.04	0.02

CI—confidence interval, bold indicates statistical significance (*p* < 0.05). AA in the respective subject groups shows a different correlation in tested variables in groups of men and women.

**Table 4:** Results of regression analysis (using the best in terms of adjusted R^2^ 4-element subset method) of AA of men and women as a function of their morpho-functional development and level of attention. Standardized Coefficients β highlighted in bold are significant with *p* < 0.05.

Test for Full Model					Standardized Coefficients β for Selected Variables							
Variable	Sex	F	*p*	Adjusted R^2^	Age	Body Height	Body Mass	BMI	CTT-1	CTT-2	Hand Grip	Shuttle Run 10 × 5 m
Civil subjects (humanities and social sciences)	w	**4.32**	**0.0082**	**0.307**		0.135			**0.425**	−0.379	**0.459**	
	m	1.90	0.1113	0.018	0.133	0.116			−0.072		−0.074	
Civil subjects (science and natural sciences)	w	**6.70**	**0.0008**	**0.432**	0.195	**−0.465**	**0.488**				**0.365**	
	m	1.33	0.2590	0.007		−0.068			−0.075	0.070	0.123	
Military subjects (theoretical)	w	**5.14**	**0.0035**	**0.355**		**2.181**	**−2.855**	**2.191**			**0.674**	
	m	**4.41**	**0.0020**	**0.065**		**1.280**	**−1.793**	**1.554**			**−0.198**	
Military subjects (practical)	w	1.28	0.3016	0.037		1.313	−1.794	1.309		−0.248		
	m	1.80	0.1306	0.016	0.059	0.128	0.060			−0.048		

Adjusted R^2^—coefficient of determination, β significant at *p* < 0.05—marked in bold, w—women, m—men.

## Data Availability

The datasets used and/or analysed during this study are available from the corresponding author on reasonable request.
